# Antimicrobial activity of methanolic extracts of *Vernonia cinerea* against *Xanthomonas oryzae* and identification of their compounds using *in silico* techniques

**DOI:** 10.1371/journal.pone.0252759

**Published:** 2021-06-14

**Authors:** Tushar Joshi, Satish Chandra Pandey, Priyanka Maiti, Manish Tripathi, Ashutosh Paliwal, Mahesha Nand, Priyanka Sharma, Mukesh Samant, Veena Pande, Subhash Chandra

**Affiliations:** 1 Department of Biotechnology, Bhimtal Campus, Bhimtal, Kumaun University, Uttarakhand, India; 2 Cell in Molecular Biology Laboratory, Department of Zoology, Soban Singh Jeena University, Almora, Uttarakhand, India; 3 Centre for Environmental Assessment & Climate Change, G. B. Pant National Institute of Himalayan Environment, Kosi-Katarmal, Almora, Uttarakhand, India; 4 Computational Biology & Biotechnology Laboratory, Department of Botany, Soban Singh Jeena University, Almora, Uttarakhand, India; 5 Environmental Information System on Himalayan Ecology, G.B. Pant National Institute of Himalayan Environment, Kosi-Katarmal, Almora, Uttarakhand, India; 6 Department of Botany, Kumaun University, D.S.B Campus, Nainital, Uttarakhand, India; South China Agricultural University, CHINA

## Abstract

Bacterial Leaf Blight (BLB) disease is an extremely ruinous disease in rice, caused by *Xanthomonas oryzae* pv. *oryzae* (*Xoo*). Although various chemicals are available to manage BLB, they are toxic to the environment as well as humans. Hence there is a need to develop new pesticides as alternatives to hazardous chemicals. Therefore, a study was carried out to discover new potent natural pesticides against *Xoo* from different solvent extracts of *Vernonia cinerea*. Among all the fractions, the methanolic extract showed the highest inhibition zone. Further, to gain mechanistic insight of inhibitory action, 40 molecules of methanolic extracts were subjected for *in silico* study against two enzymes D-alanine—D-alanine ligase (Ddl) and Peptide deformylase (PDF). *In silico* study showed Rutin and Methanone, [1,4-dimethyl-7-(1- methylethyl)-2- azulenyl]phenyl have a good binding affinity with Ddl while Phenol, 2,4-bis(1-phenylethyl)- and 1,2-Benzenedicarboxylic acid, diisooctyl ester showed an excellent binding affinity to PDF. Finally, the system biology approach was applied to understand the agrochemical’s effect in the cell system of bacteria against both the enzymes. Conclusively, these four-hit compounds may have strong potential against *Xoo* and can be used as biopesticides in the future.

## Introduction

The global rice demand was estimated to rise from 439 million tons (milled rice) in 2010 to 496 million tons in 2020 and further, it will increase to 555 million tons in 2035 as per need of increasing the population, according to the Food and Agricultural Policy Research Institute (FAPRI) (http://ricepedia.org/rice-as-a-crop/rice-productivity) (accessed date- 6 April 2021). The crop is widespread all over the world due to its broad adaptability under different environmental conditions and also an important food crop as a huge chunk of the global population depends on rice for its nutrition. But, the current day the crop is facing a huge amount of yield losses throughout the world due to a disease named Bacterial Leaf Blight (BLB) caused by a bacteria known as *Xanthomonas oryzae* pv. *oryzae* (*Xoo*) [1]. The disease commonly causes significant yield losses of 20–30% or even up to 80% at the maximum tillering stage. At the booting stage of the plant, the *Xoo* lowers the quality of grains and causes broken kernels. Rice plants in both tropical and temperate environments, particularly in irrigated and rainfed lowland areas, areas that have weeds and stubbles of infected plants with optimum temperature 25−34°C and relative humidity above 70%are prone for the disease.

In India, BLB disease occurs from north to west and east to south with approximate yield losses up to 60–80% [2]. This significant loss affects the farmer’s fields along with the industries, and the global population depends on the various product of rice. Therefore, the management of BLB disease is a necessary step towards the protection of rice crops worldwide. Studies showed three different approaches, *viz*, biological control, chemical control, and genetic resistance, are applied for the management of BLB disease [3, 4]. Among these methods, the biological control method is one of the most appropriate for the above disease management, as it is environment friendly and non-toxic not only for humans but also small microorganisms to large mammals [5]. In some of the studies, researchers reported the use of plant growth-promoting rhizobacteria to manage the BLB disease [6]. Several phytochemical extracts from plant species including *Ocimum gratissimum*, *Curcuma longa*, *Acoruscalumus*, *Tamarindus indica*, *and Azadirachta indica*, have been reported for the management of BLB disease [7–9]. Previously many technologies have been used to protect rice crops from BLB however, directly targeting the bacterial growth enzymes is an efficient strategy to stop BLB. In this regard, Ddl and PDF are key bacterial growth enzymes. Ddl is an ATP-dependent bacterial enzyme that plays a vital role in the intracellular stages of peptidoglycan biosynthesis [10]. The main product of the Ddl enzyme is D-alanyl-D-alanine, which is the terminal dipeptide of UDP-N- acetylmuramoylpentapeptide [11] and is eventually involved in trans-peptidation. Whereas PDF is important for bacterial cells and catalyzes the removal of the N-formyl group from N-terminal methionine. Hence, Ddl and PDF enzymes have elected the targets for the present study. Thus, due to the importance of BLB disease of rice, the present study aims to find out the effective natural pesticide compounds from *Vernonia cinere* against *Xanthomonas oryzae* pv. *oryzae* disease.

The plant *Vernonia cinerea* is used in the component of dasapushpam (in Sanskrit, dasa ¼ ten and pushpam ¼ flowers), an herbal combination of 10 plants that are traditionally used in the Kerala state of India. It is known as purple fleabane and Sahadevi and has been reported to use in traditional medicine for treating various ailments including inflammation, diarrhea, cough, smoking cessation, asthma, Parkinson’s disease, leprosy, and conjunctivitis [12]. The plant has also been reported to show several pharmacological properties including analgesic, anti-inflammatory, antipyretic, antibacterial, anti-oxidant, anti-glycemic, antidiarrhoeal, antitumor, antiplasmodial, and anti-Helicobacter pylori activity. In the current work, four different solvent extracts of the whole plant were tested against *Xoo* bacterium under in-vitro conditions for their anti-BLB activity. Further, Ddl and PDF target-specific phytochemicals were identified against the bacteria by molecular docking, molecular dynamics simulation, and system biology. Hence, in this study, we have evaluated the anti-BLB activity of *V*. *cinerea* extracts in vitro conditions and identified potential natural agrochemicals targeting two key enzymes, i.e., Ddl, and PDF by *in silico* approaches. Further system biology approach was applied to understand the possible mechanism of action of these target-specific drug molecules. The network analysis was also conducted to identify key reactions in the *Xoo* system that could affect cellular reactions after drug interaction.

## Material and methods

### Plant collection and extraction

The whole plant of *V*. *cinerea* was collected from District SantKabir Nagar, Uttar Pradesh (specific permission was not required because this plant is wild and can found anywhere). Identification of plant was done by Forest Research Institute, Dehradun. Further, the collected plant was dried and pulverized by using a mechanical grinder to a coarse powder, and the powder was sifted and stored in airtight containers and kept at room temperature until processing. The preparation of crude extract was done with the help of the Soxhlet apparatus. Four solvents; Methanol, Ethanol, Chloroform, and Chloroform+Ethanol were used in 90% (v/v) concentration for extraction of phytochemicals of *V*. *Cinerea*. 100 g dry powder of *V*. *cinerea* was dipped in four different solvents combinations for 24 hrs. The extracts were filtered and concentrated under reduced pressure and the remaining solvents were evaporated. Before using, the dried extracts of plant material were dissolved in 0.7% DMSO (Dimethyl sulfoxide) and stored at 4°C.

### Antibacterial activity of *V*. *cinerea*

*Xoo* was purchased from the Indian Agriculture Research Institute–ICAR Delhi. The antimicrobial activity of plant extracts was analyzed on agar plates, liquid media, and MIC was calculated according to the modified laboratory protocol of the Clinical and Laboratory Standards Institute [13]. Briefly, 0.1 mL bacterial culture was inoculated on Peptone-sucrose (PS) agar plates and incubated at 28°C with filter discs (5 mm diameter) saturated with different dilutions of plant extracts (25, 50, and 100 μg/mL) for one day. The inhibition zones (mm) were measured by determining the diameter of the clear area. Similarly, the antibacterial activity was also measured by incubating the above-mentioned concentrations of plant extracts into PS broth media at 28°C for 24hours. For the MICs, different concentrations (2.5, 5, 10, 25, 20, 50, and 100 μg/mL) of plant extracts were added to *Xoo* culture in PS media and incubated for24 h at 28°C. The lowest concentration of plant extracts that prevented microbial growth (showed no turbidity) was measured by spectrophotometer at OD 600. Each test was performed in triplicate. Tetracycline was employed as a positive control. Cultures without plant extracts or antimicrobials were used as a negative control.

### Statistical analysis

For statistical analysis, all experimental data were analyzed in triplicates, and results were calculated as mean±SD. The results (pooled data of three experiments) of experiments were calculated by one-way ANOVA. All analysis was done by using Graph Pad Prism (version 3.03) software.

### *In silico* screening

#### Phytochemicals library and ligand preparation

To know the insight activity of methanol extracted compounds of *V*. *cinerea* against *Xoo*, a library of 40 phytochemicals was constructed by using text mining analysis. Several studies have been reported on GC-MS analysis of phytochemicals of different extracts of *V*. *cinerea* and from these studies, we collected the phytochemicals found in methanolic extract and constructed a library [14, 15] by downloading 3D structures of these phytochemicals from PubChem in SDF format, and converted into PDB files using Open Babel. The Reference molecule ANP (Phosphoaminophosphonic acid-adenylate ester) and 56V [(3R)- 2,3-dihydro[1,3]thiazolo [3,2 a]benzimidazol-3-ol)] which was co-crystallize with Ddl and PDF proteins, were downloaded from Protein Data Bank.

#### Protein preparation

The 3D structures of Ddl (PDB ID 4L1K) and PDF (PDB ID 5CY8) enzymes of *Xoo* (Xoo1075) were retrieved from the Protein Data Bank (https://www.rcsb.org). In the protein preparation, all water molecules, ions, and ligands were removed using PyMOL software. After that, the addition of hydrogen atoms to the receptor molecule was carried out by MGL Tools. The preprocessed structures of both proteins were then saved in PDB format for further analysis.

#### Molecular docking and visualization

The docking calculation process was performed to obtain a population of possible orientations and conformations for the ligand at the binding site by using PyRx open-source software (GUI version 0.8 of AutodockVina). This software performs the prediction of the bound conformation based on the binding affinity. The grid center for docking was set for Ddl was X = 12.19, Y = 16.17, and Z = 17.76 and for PDF was X = 7.589, Y = -15.291, and Z = -2.049, and the dimensions of the grid box were set as 25.00 × 25.00 × 25.00 Å having a spacing of 0.375 Å between the grids points. Molecular docking of compounds was performed in the active site of Ddl and PDF proteins. Further, Molecular interactions between protein-ligand complexes, including hydrogen bonds and the bond lengths, were visualized using Ligplot+ v.1.4.5 software.

#### Molecular dynamics (MD) simulations

MD simulations were carried out with docked complexes using a GROMACS 5.0 package and the topologies files were generated using the CHARMM 36 force field. Afterward, complexes were solvated with a water model followed by neutralization by adding the ions. After adding ions, to relax the structure, Energy minimization was performed at 10 KJ/mol with the steepest descent Algorithm by using Verlet cut off-scheme and the total nsteps of protein and protein-ligand complex energy minimization cycle was 50,000. The equilibration step was performed in NVT (constant volume) as well as NPT (constant pressure) ensemble conditions, each with a 100 ps time scale. Further, the production of MD simulation was performed at a constant temperature of 300 K and a constant pressure of 1 atm with a time step of 2 fs, using the Parrinello-Rahman for constant pressure. The final MD simulations were produced using the LINCS algorithm for a 100 ns time scale. The generated trajectories were used to analyze the behavior of each complex in the explicit water environment. The deviations of the protein-ligand complex system were calculated by using Root mean square deviation (RMSD), Root mean square fluctuation (RMSF), Radius of gyration (RG), interaction energy. The short-range Lennard-Jones energy model was used for the calculation of interaction energy between proteins and compounds.

#### System biology analysis model construction

To analyze the effect of different amounts of the agrochemical against two Ddl and PDF in the bacterial cell system, a detailed analysis of the system biology of the bacterial cell was performed. For that, a model of the integrated biochemical levels was developed to understand the reaction mechanism in which these enzymes work as catalyzing agents. For the construction of biological networks, a System Biology Graphical Notation (SBGN) was used to represent cellular components (S1 Fig in S1 File) [16]. The pathway of enzyme reactions was constructed using Cell Designer 4.1 and stored in Systems Biology Markup Language (SBML). SMBL is a machine-readable expression for reflecting the biological networks. System biologists use existing signs of DNA, RNA, protein, simple molecule, catalysis, stimulation, inhibition, phosphorylation, activation, and degradation to develop the pathway by using Cell Designer software to study live cells on a computer. In a cell designer, simulation can also be conducted by utilizing SBMLODE Solver and Copasi.

#### Kinetic rate equations assignment

To generate the kinetic rate equations for every reaction in the model, SBML squeezer version 2.1 of Cell Designer was used. SBML squeezer makes the whole process error-free and easy through Cell Designer. Rate laws comprising different forms of general mass action and enzyme kinetics were also generated by the SBML squeezer (S3 Table in S1 File). Kinetic rate equations for various enzymatic reactions include Michaelis–Menten kinetics and hill equation for single substrate reactions, irreversible non-modulated non-interacting reactant and enzymes, bi-uni enzyme reactions, bi–bi enzyme reactions, thermodynamics, and kinetic modular rate laws [17, 18].

#### Model simulation

SBMLbODE Solver Library (SOSlib) of cell designer was employed to simulate the dynamic nature of the developed model. This library allows the management of ordinary differential equations (ODE) based simulations that are common procedures for quantitative investigation of biological networks. SOSlib is a programming library commonly used in the symbolic and numerical interpretation of biochemical reaction network models encoded in the SBML. While working with biological networks, relevant parameters are needed to be evaluated for their dependency on other elements of the model. The native library was run by a simulation engine and obtained results are shown in a Graphical User Interface (GUI) window written in the JAVA programming language [17].

#### Network analysis

Reaction models of Ddl and PDF enzymes were built by using Cell Designer and exported in SBML format to Cytoscape 2.8.3 software by using Biological Network Manager (BiNoM). BiNoM is a Cytoscape plugin to assist numerous biological network activities that are demonstrated in common systems biology formats (SBML, SBGN, BioPAX). It is also used to undertake investigations of the complicated network structure. Various types of plugins are present in Cytoscape software, which enable us to decode the complications of biological networks. Network Analyzer plugin was used to investigate and visualize the crucial elements in enzyme reaction in our model [18].

## Results

### Antimicrobial activity of *V*. *cinerea*

The antibacterial potential of *V*. *cinerea* was evaluated according to their zone of inhibition against *Xoo*. The zones of inhibition were compared with the activity of the standards antibiotic Tetracycline as positive control while DMSO was used as a negative control (S1A, S1B, and S1C Fig in S1 File).

It was observed that the methanolic extract of the plant was the most effective among the three solvent extracts (Ethanol, Chloroform, and Chloroform+Ethanol) and showed a better zone of inhibition at different concentrations (25, 50, and 100 μg/ml). The Chloroform, Ethanol, and Chloroform+Ethanol extracts showed no zones of inhibition at low concentrations therefore these extracts were omitted for further study.

The methanolic extract inhibited the growth of *Xoo* bacteria after 24 h of incubation and showed a remarkable zone of inhibition. At the concentration, 25 μg/ml the size of the zone was 16.0±1.0 mm, while at 50 μg/ml concentration, the size of the zone was 18.1±1.0 mm and at 100 μg/ml concentration, the size of the zone was 22.6±2.08 mm as compared to positive control tetracycline (33.17±3.7mm at 5 μg/ml) (S1 Table in S1 File). The methanolic extract showed significant (P < 0.001) growth inhibition activity up to 24 h.

**Fig 1:**
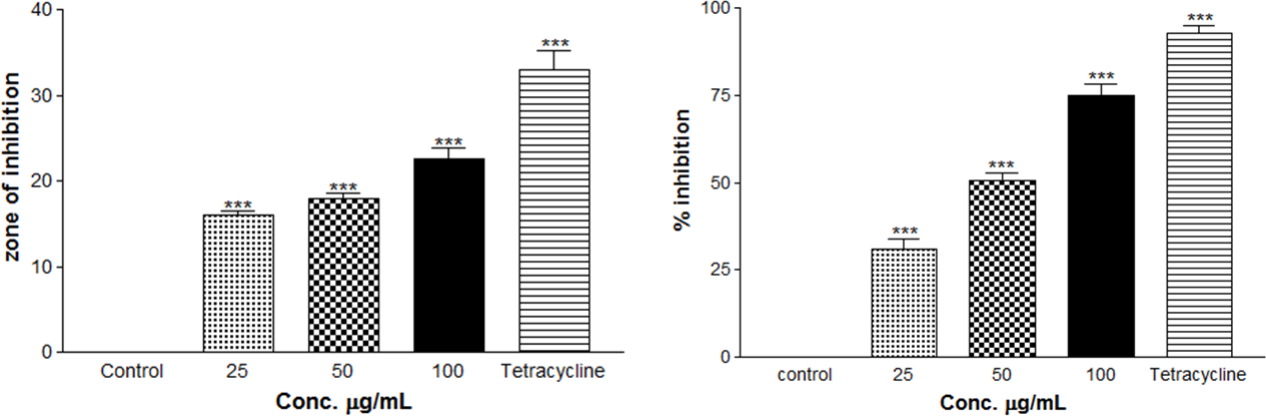
The *in vitro* antibacterial activity of different concentrations of whole plant extract of *V*. *cinerea* against *Xanthomonas oryzae* pv.*oryzae* (*Xoo*). (A) Bar diagrams showing zone of inhibition at the concentration of 25, 50, and 100 μg/mL (B) and % inhibition of *Xanthomonas oryzae pv oryzae* (*Xoo*) after incubation with different concentration of test compounds or the positive control tetracyclin (1 μg/mL) for 24 h. There were three replicates in each experiment, and the data are the mean±SD for each concentration. Significance values indicate the difference between the untreated groups and treated groups with various concentrations of plant extract or tetracycline (***, p<0.001).

In liquid culture medium, the treatment of *Xoo* at the concentration of 25μg/ml showed 31.14±0.54% inhibition while at the concentration 50 μg/ml, the percent inhibition was 50.50±0.89% and at the concentration100 μg/ml the percent inhibition was 74.94±0.78% as compared to positive control tetracycline (92.61±0.80) (S1 Table in S1 File). Thus, the methanolic extracts showed significant (P < 0.001) growth inhibition activity up to 24 h (Fig 1B). The methanolic extracts showed significant inhibitory effects within the range of MIC. The MIC value of *V*. *cinerea* extract for *Xoo* was measured to be 10 μg/ml as compared to 1 μg/mL (tetracycline) as a positive control.

In addition, *in silico* studies were carried out to evaluate the mechanism of the phytochemicals of methanolic extracts and a compound’s library was prepared. Further, these molecules were subjected to molecular docking and dynamic simulation study against two important proteins of *Xoo viz*., Ddl and PDF.

### Molecular docking analysis

**Fig 2:**
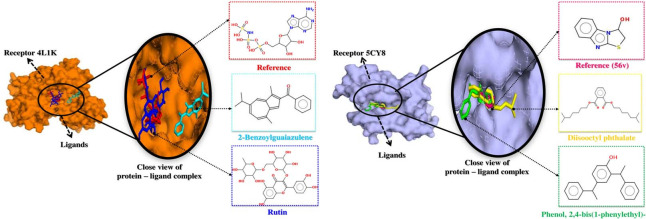
3D view of the docked ligands position in the crystal structure of proteins (A) Ddl (B) PDF and 2D structure of screened compounds.

The library of 40 compounds was subjected for molecular docking against the Ddl and PDF receptor. Out of 40 compounds, the top 2 compounds namely; rutin (-8.3 kcal mol^-1^) and Methanone, [1,4- dimethyl-7-(1- methylethyl)-2-azulenyl]phenyl- (-7.8 kcal mol^-1^) showed a binding affinity with Ddl and Phenol, 2,4-bis(1-phenylethyl)- (-7.5 kcal mol^-1^) and 1,2-Benzenedicarboxylic acid, diisooctyl ester (-7.4 kcal mol^-1^) showed their binding affinity with PDF. These compounds showed better and significant binding energy with Ddl and PDF as compared to reference molecule ANP (-8.3 kcal mol^-1^) and 56V (-7.7 kcal mol^-1^). The hydrogen and hydrophobic bonds between protein and ligands have been shown in Table 1 and the binding of ligands to the protein has been shown in Fig 2A and 2B in the 3Dmodel.

**Table 1:** Binding affinity of methanolic extract compounds against Ddl and PDF enzymes.

S. No.	Phytochemicals and Protein name	Binding Energy (Kcal/mol)	Hydrogen Bonds	Hydrophobic Bonds
1	ANP-**Ddl (Ref)**	-8.3	Lys141, Asp302, Glu315, Asn314, Glu221, Ala222, Val224, Lys185	Val195, Ser191, Ser192, Glu228, Phe304, Ala223, Phe183
2	Rutin-**Ddl**	-8.3	Val327, Asn317, Glu16, His107, Glu112, Gln189, Glu315, Asp302, Glu230, Arg300, Gly321	Pro320, Ser326, Val19, Leu319
3	Methanone, [1,4- dimethyl-7-(1- methylethyl)-2- azulenyl]phenyl- -**Ddl**	-7.8	Asn314	Glu221, Phe304, Phe183, Val224, Gly226, Glu228, Val195, Ser192, Lys185
4	56V-**PDF (Ref)**	-7.7	Glu142, Tyr69, Gly98	Gly46, Val45, Leu100, Arg137, Glu97, Phe134, Val138, His141
5	Phenol, 2,4-bis(1- phenylethyl)- **PDF**	-7.5	Leu105	Arg106, Asp164, Arg68, Cys99, Leu100, Gly44, His43, Val145, Glu142, His141, Gly98, Tyr69
6	2,4-bis(1- phenylethyl)- and 1,2- Benzenedicarboxylic acid, diisooctyl ester- **PDF**	-7.4	Cys99, Gly104	Pro103, Leu100, Ile5, Leu105, Val45, His141, Phe134, Val138, Arg137, Glu97, Tyr69, Trp96, Arg106, Gly98, Arg68, Asp164

### Molecular dynamic (MD) simulation analysis

To analyze the stability of four compounds with two proteins Ddl and PDF, MD simulation was conducted for protein-ligand complexes. Four compounds namely; rutin and Methanone, [1,4- dimethyl-7-(1- methylethyl)-2-azulenyl]phenyl- with Ddl and Phenol, 2,4-bis(1-phenylethyl)- and 1,2-Benzenedicarboxylic acid, diisooctyl ester with Peptide deformylase, which possessed the best binding energies were subjected for MD simulation. The structural changes in protein-ligand complex and dynamic behavior patterns were analyzed by the RMSD, RG, interaction energy, and RMSF calculations.

### Root Mean Square Deviation & radius of gyration

**Fig 3:**
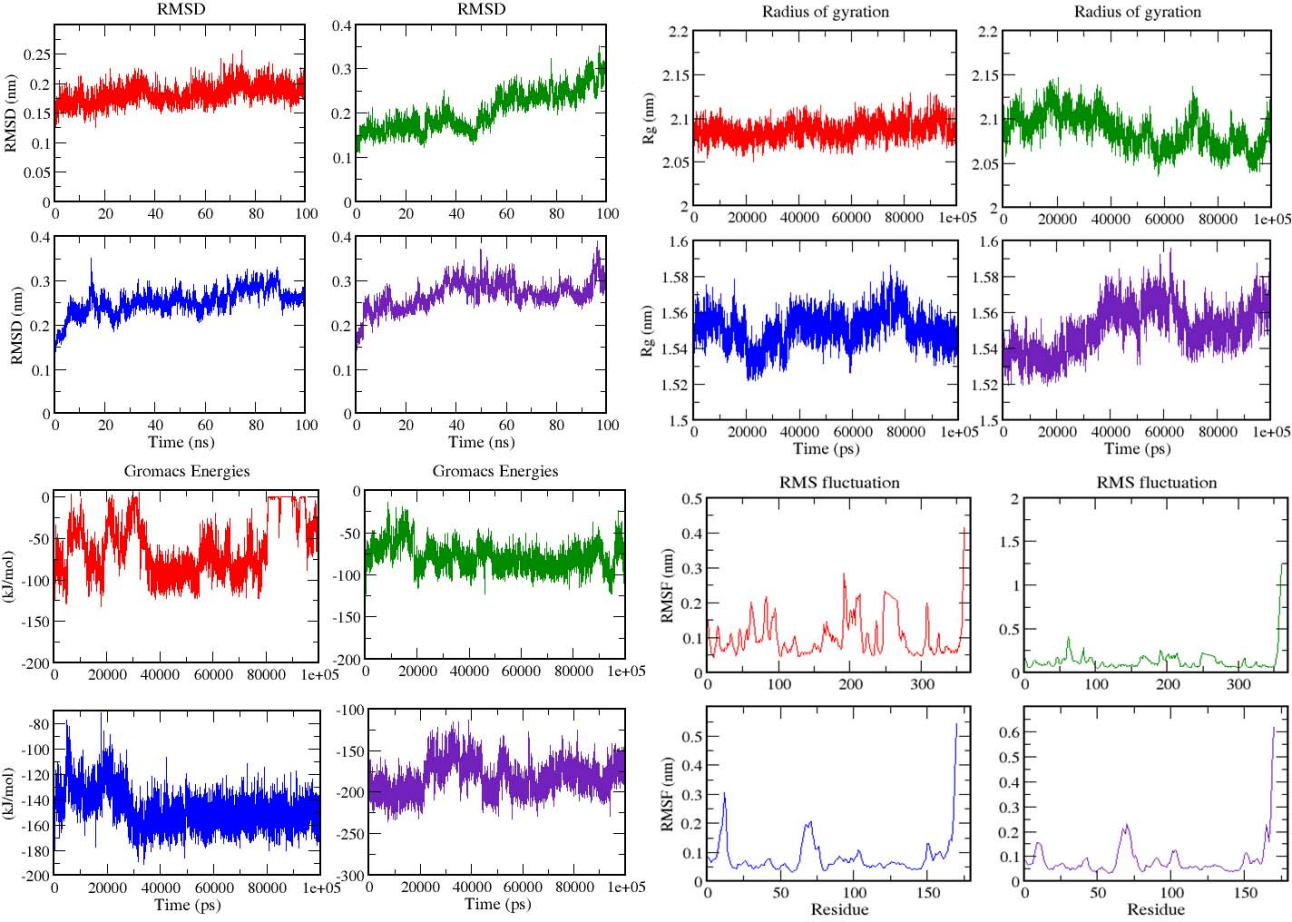
MD simulation analysis of proteins-ligand complexes for 100 ns analysis **(A)** RMSD **(B)** Rg **(C)** interaction energy **(D)** RMSF. The color code for all panels are Rutin-Ddl (Red), Methanone, [1,4-dimethyl-7-(1- methylethyl)-2-azulenyl]phenyl- -Ddl (Green), Phenol, 2,4-bis(1-phenylethyl)- PDF (blue), 2,4-bis(1-phenylethyl)- and 1,2-Benzenedicarboxylic acid, diisooctyl ester-PDF (indigo).

Root Mean Square Deviation (RMSD) was analyzed to calculate the deviation among protein-ligand complexes during the 100 ns MD simulation to understand the effect of ligands on protein dynamics. Fig 3A depicts the RMSD plot of protein-ligand complexes (Rutin-Ddl, Methanone, [1,4-dimethyl-7-(1- methylethyl)-2-azulenyl]phenyl- -Ddl, Phenol, 2,4-bis(1- phenylethyl)- PDF, and Benzenedicarboxylic acid, diisooctylester-PDF). In the case of the complex system, all complexes are showing stability with protein in 100 ns simulation. The average value of RMSD is 0.18 nm (red), 0.20 nm (green), 0.25 nm (blue) and 0.26 nm (indigo) (Table 2). Further, The Radius of Gyration (Rg) was calculated to analyze the stably folded or unfolded protein and complexes system. The average Rg value of complexes is 1.79±0.077 nm (red), 1.79±0.080 nm (green), 1.33±0.054 nm (blue), and 1.33±0.053 nm (indigo) (Table 2) (Fig 3B).

**Table 2:** RMSD, Rg, and interaction energy value of screened compounds.

S. No.	Phytochemicals and Protein name	Average RMSD (nm)	Average Rg (nm)	Interaction Energy (kJ mol^-1^)
**1**	Rutin-**Ddl**	0.18±0.013	1.79±0.077	-60.75
**2**	Methanone, [1,4- dimethyl-7-(1- methylethyl)-2- azulenyl]phenyl- -**Ddl**	0.20±0.039	1.79±0.080	-78.95
**3**	Phenol, 2,4-bis(1-phenylethyl)- **PDF**	0.25±0.022	1.33±0.054	-148.115
**4**	Benzenedicarboxylic acid, diisooctyl ester-**PDF**	0.26±0.024	1.33±0.053	-182.885

### Calculation of interaction energy

The average interaction energy of compounds with PDF was better than the Ddl enzyme. The interaction energy of Phenol, 2,4-bis(1-phenylethyl) (blue), and Benzene dicarboxylic acid, diisooctyl ester (indigo) showed -148.115kJ mol^-1^ and -182.885 kJ mol^-1^ respectively with PDF enzyme while compounds Rutin (red) Methanone, [1,4-dimethyl-7-(1- methylethyl)-2- azulenyl]phenyl- (green) showed -60.75 kJ mol^-1^ and -78.95 kJ mol^-1^ respectively with Ddl enzyme (Table 2) (Fig 3C).

### Calculation of residual components fluctuation

The Root Mean Square Fluctuation (RMSF) calculation was performed to know the fluctuation of residues in protein-ligand complexes during the 100 ns trajectory period and a graph was plotted to compare the flexibility of each residue in the complex. The maximum fluctuations in rutin during the simulation were 0.28 nm in SER192 residue followed by 0.24 nm in Val193and Gly194 (shown in red color) and Methanone, [1,4-dimethyl-7-(1- methylethyl)-2- azulenyl]phenyl- showed maximum fluctuation of 0.41 nm in Asp62 and little fluctuation was recorded between 61 to 66 and 190 to 193 residues with Ddl enzyme which is shown in green color. Compounds with PDF enzyme showed very little fluctuation in residues. Phenol, 2,4- bis(1-phenylethyl) showed fluctuation in only one residue Arg12 i.e., 0.30 nm (blue). Benzenedicarboxylic acid, diisooctylester is depicted in indigo color and it showed fluctuation between 67 to72 number residues (Fig 3D).

### System biology analysis

#### Constructed model description

**Fig 4:**
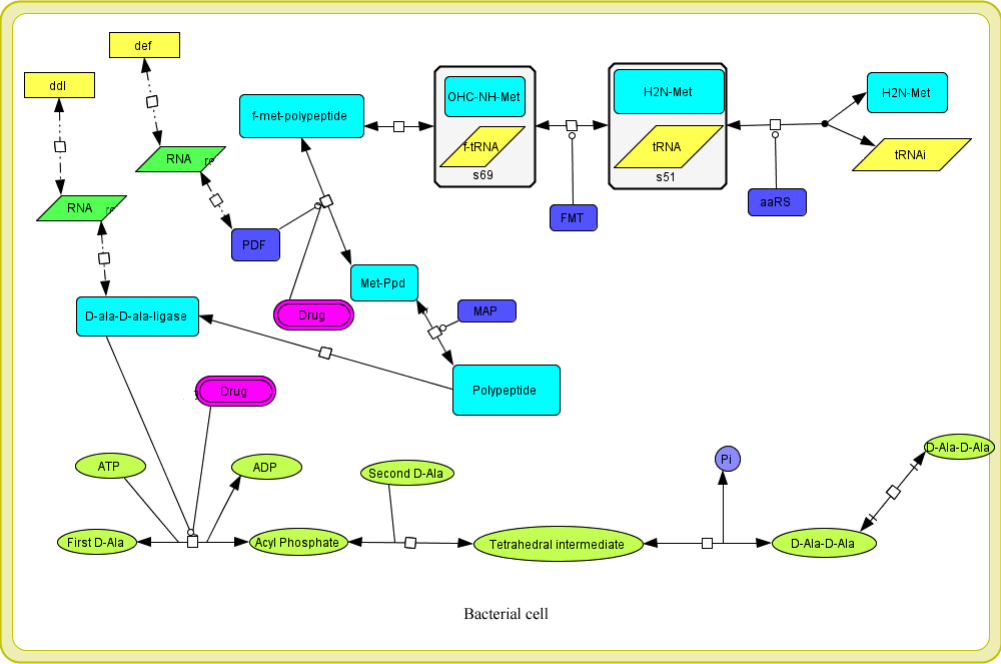
Drug activity on Ddl and PDF enzymes was constructed using cell designer in bacteria, and its effect the production of enzymes productivity. The diagram is showing the simple molecules, proteins, mRNA, DNA, enzymes, catalysis, and inhibition, etc.

A model was constructed to understand the mechanism of the agrochemical in a bacterial cell against Ddl and PDF enzymes. This model aimed to get the information of agrochemical reaction inside the bacterial cell. The pathway of these two enzymes was constructed in this model in which Ddl and PDF enzymes play their essential role. The first pathway of the model shows the PDF enzyme removes N-formyl group in the methionine cycle to form a mature protein. In this pathway, we showed the agrochemical inhibition on the PDF enzyme. The second pathway was for the Ddl enzyme, which is used as a catalyst for the formation of D-alanyl-D-alanine for the biosynthesis of the bacterial cell wall. In this pathway, we applied the agrochemical inhibition on the Ddl enzyme. Both models were constructed using preciously available literature describing the mechanism of these two pathways [19, 20]. The model comprises one compartment, 25 species, two genes, 4 RNA, ten proteins, and 14 reactions (Fig 4).

### Dynamic behavior of an agrochemical on PDF and Ddl

**Fig 5:**
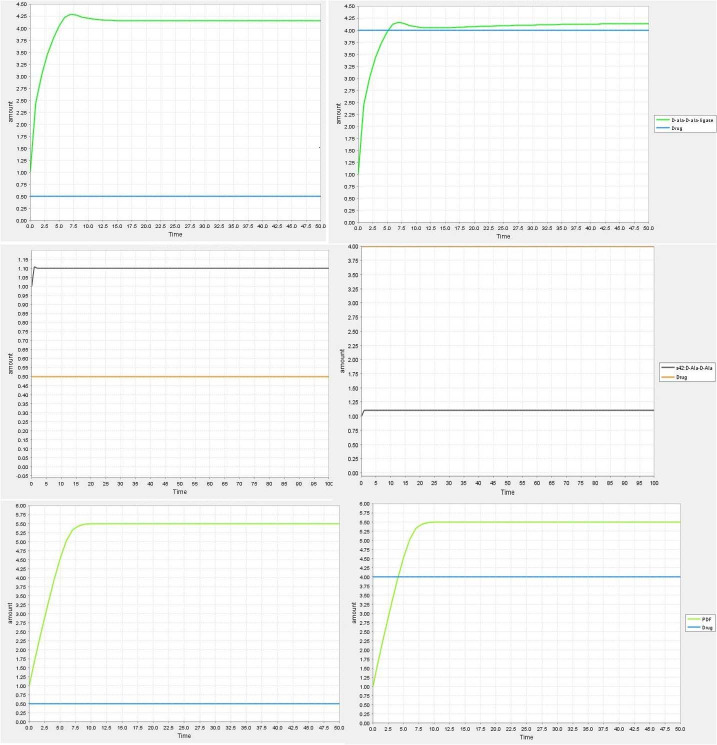
Dynamic behavior analysis of drug inhibition activity on different amounts with (A) Ddl (B) D-alanyl-D-alanine and (C) PDF (amount 1.0 and 4.0).

Simulation analysis of agrochemical was carried out to analyze the inhibition effect of agrochemical on enzymes through an integrated systems biology approach. For the prediction of the dynamic behavior of the reaction, SBML squeezer was employed to produce the rate laws. The predicted dynamic behavior can be used to understand the effect of the agrochemical on Ddl and PDF enzymes. The value for each molecular species ranges from 1 to 6.0 (S2 Table in S1 File). The gene, protein, and mRNA values were set at 1.0 due to the basal amount in bacterial cells [21]. The agrochemical amounts were set at 0.5, 1.00, 1.5, 2.00, 3.00, and 4.00 and calculate the enzyme’s productivity on its products. The graph shows that as the amount of the agrochemical was increased, the amount of the Ddl enzyme was decreased (Fig 5A and S3A Fig in S1 File). However, the amount of D-Ala-D-Ala fluctuated very little as the amount of the agrochemical increased only in one amount of agrochemical, the production of D-Ala-D-Ala was found to be decreased (Fig 5B and S3B Fig in S1 File), but the amount of PDF remained the same in every amount of agrochemical (Fig 5C and S3C Fig in S1 File).

### Network analysis of enzyme pathways

**Fig 6:**
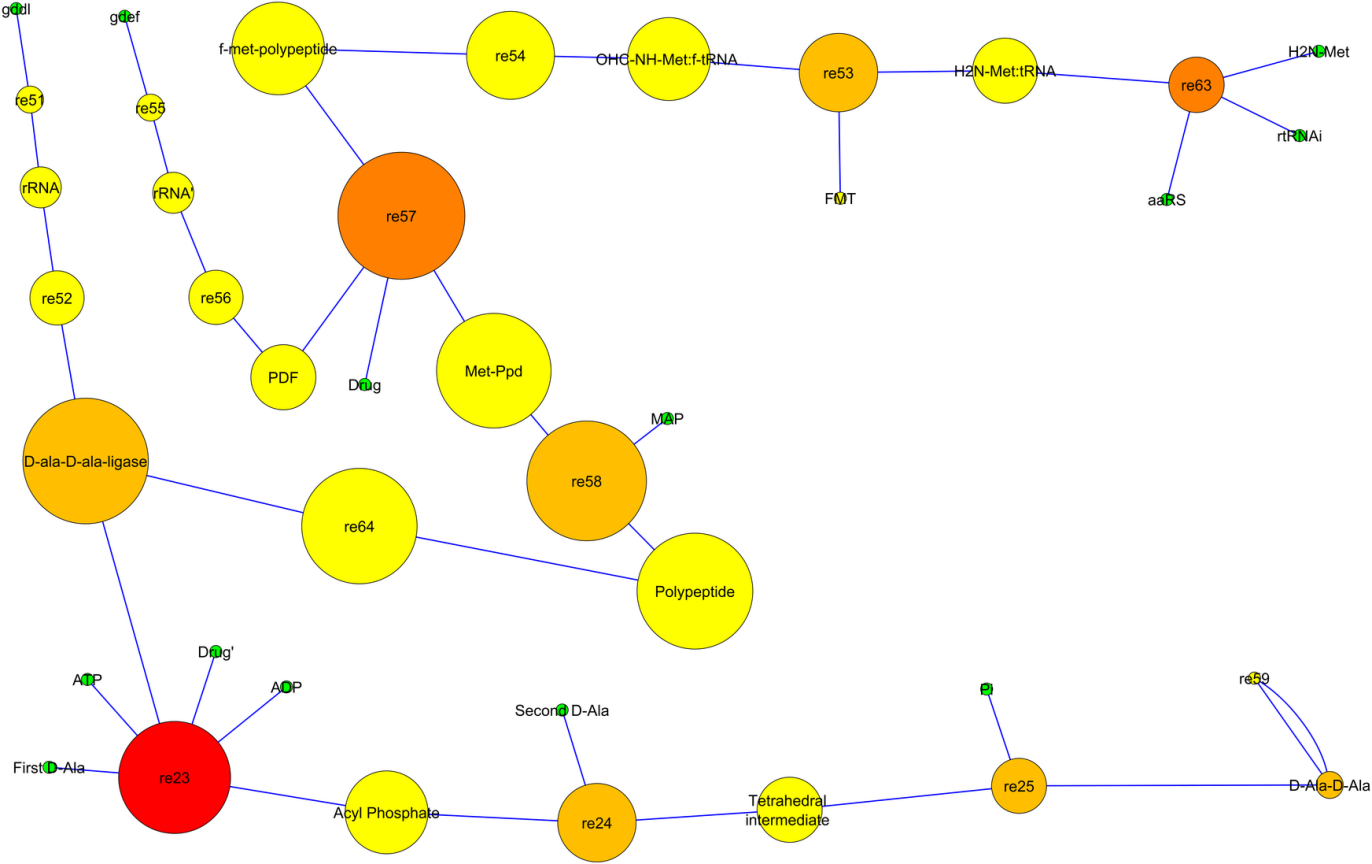
Mapping of drug activity network: Visualization of complete network pathway to map hub nodes.

The interactive network of enzyme inhibition and their effect on their product was found to have 40 nodes and 40 edges. All the data were analyzed as scale-free property as predicted from the system biology network [22, 23]. Enzyme pathway and agrochemical inhibition network were utilized to estimate the average path length. For network analysis, sample parameters were used, which are shown in S4 Table in S1 File. The node size “Degree” and node color “Betweenness Centrality" can be mapped using visual style and may be employed to determine hub nodes in the cell designer. The Betweenness Centrality of each node represents the amount of control that any node imposing over the interactions of other nodes in the network [24] and it is a number between 0 and 1 [18]. The nodes presented in red color are hub nodes and maybe a significant regulatory character in enzyme inhibition (Fig 6). The nodes represented in yellow color show the moderate regulatory effect on enzyme pathways.

## Discussion

In modern agriculture, natural products are being exploited as biocontrol agents because they are eco-friendly and present a sustainable approach to disease control of crops. In India, in terms of production and use, rice is the second-largest cereal crop, and a lot of chemical fertilizers, pesticides, fungicides, and bactericides are applied to keep the plant healthy disease-free. All these chemicals are very harmful to the environment [25] and keeping this problem in mind, this study was carried out to evaluate the antibacterial activity of *V*. *cinerea* extracts against *Xoo*.

The plant *V*. *cinerea* is well established for its anti-bacterial properties against many types of bacteria [26–28] (Table 3). In the present work, we also evaluated this plant against *Xoo*. The bacterial inhibitory percentage in 25, 50, and 100 μg/ml concentration revealed that the *V*. *cinerea* is very effective against *Xoo*. The MIC value (10 μg/ml) was also found to be significant in terms of the inhibition zone. The results of our study show similarity with the previously reported activity of *V*. *cinerea* against several bacteria *viz*., the methanolic extract showed 3.13 mg/ml MIC value against gram-negative bacteria *P*. *aeruginosa* [29], and silver nanoparticles made by leaf extract of *V*. *cinerea* showed 80 μg/mL MIC value against *Xanthomonas campestris* pv. *Malvacearum* [30]. The MIC of plant extract in a study was high (3.13mg/mL and 80 μg /mL) against both gram-negative bacteria as compared to our study (10 μg/ml) and it shows that *V*. *cinerea* methanolic extract has the potential to inhibit a wide variety of bacteria in low concentration.

**Table 3:** The activity of *V*. *cinerea* plant extract against different bacteria.

**Plant Name**	**Bacterial Pathogen**	**Extracts showing Antibacterial activity**								**References**
*Vernonia cinerea*(L).		VNH	VCH	VEA	VCME	PECV	EECV	Benzene	AgNO_3_	
	*Escherichia coli*	**+**	**+**	**+**	*	**+**	**+**	**+**	*	Sonibare et al., 2016, Somasundaram et al., 2010, Gupta et al., 2003 [26–28]
	*Pseudomonas aeruginosa*	**+**	**+**	**+**	**+**	**+**	**+**	**+**	*	Sonibare et al., 2016, Somasundaram et al., 2010 Gupta et al., 2003, Latha et al., 2010 Gupta, 2003 [26–29]
	*Proteus vulgaris*	**+**	**+**	**+**	*	*	*	*	*	Sonibare et al., 2016 [28]
	*Klebsiella pneumonia*	**+**	**+**	**+**	*	**+**	**+**	*	*	Sonibare et al., 2016, Somasundaram et al., 2010, Gupta et al., 2003 [26–28]
	*Xanthomonas campestris* pv. *malvacearum*	*	*	*	*	*	*	*	**+**	Sahayara et al., 2015 [30]
	*Bacillus subtilis*	**-**	**+**	**+**	*	**+**	**+**	**+**	*	Sonibare et al., 2016, Somasundaram et al., 2010 Gupta et al., 2003 [26–28]
	*Staphylococcus aureus*	**+**	**+**	**+**	*	**+**	**+**	**+**	*	Sonibare et al., 2016, Somasundaram et al., 2010, Gupta et al., 2003 [26–28]
	*Aspergillus flavus*	**-**	**-**	**-**	*	*	*	*	*	Sonibare et al., 2016 [28]
	*Bacillus cereus*	*	*	*	*	**+**	+	*	*	Somasundaram et al., 2010 [27]
	*Shigella dysenteriae*	*	*	*	*	*	*	**+**	*	Gupta et al., 2003 [26]
	*Salmonella* typhi	*	*	*	*	*	*	**+**	*	Gupta et al., 2003 [26]
	*Micrococcus luteus*	*	*	*	*	*	*	**+**	*	Gupta et al., 2003 [26]
	*Staphylococcus epidermidi*	*	*	*	*	*	*	**+**	*	Gupta et al., 2003 [26]

VCME- *Vernonia cinerea* crude methanolic extract, VNH- *V*. *cinerea* n-Hexane fraction, VCH- *V*. *cinerea* chloroform fraction, VEA- *V*. *cinerea* Ethyl acetate fraction, PEVC (petroleum ether *V*. *cinerea*), EEVC (alcoholic extracts *V*. *cinerea*), AgNO_3_**(**Silver nitrate)

Sign “+” (Result positive) “–” (Result negative) “*” (Study not performed)

Through *in silico* approach, four-hit compounds were screened from the methanolic extract having an excellent binding affinity with PDF and Ddl enzymes of the bacteria. Out of four, two compounds Rutin and 1,2-Benzenedicarboxylic acid, diisooctyl ester have been reported to show antibacterial activity in previous reports against various bacteria [1, 31–33]. Two compounds Methanone, [1,4-dimethyl-7-(1-methylethyl)-2-azulenyl]phenyl- and Phenol, 2,4-bis(1-phenylethyl)- first time reported as an antibacterial against *Xoo* by *in silico* study. Moreover, these compounds’ binding affinity and RMSD were better against PDF and Ddl enzymes. The interaction energy analysis of the compounds indicates, higher interaction energy with the PDF enzyme and lower interaction energy with the Ddl enzyme compared to the average short-range Lennard-Jones energy, -99.1±7.2 kJ mol^-1^ [34]. Root mean square fluctuation analysis for residual mobility showed constituent residues’ fluctuation for each complex during 100 ns MD simulations. The fluctuation of residue describes that the ligand binding to protein is not disrupting the original conformation of residues. This result suggests that the complexes have active interaction with their residues in the context of RMSF calculation.

In addition, the system model was created to simulate the mechanism of inactivation of Ddl and PDF enzymes by agrochemical inside the bacterial cell. In the cell system, the PDF enzyme helps to remove *N*-formyl group from the enzymes [35]. In the bacterial cell, protein synthesis starts with *N* formyl methionine, and the newly synthesized polypeptide is converted to mature protein through the sequential removal of the *N*-formyl group and methionine [36]. The graph (Fig 5A) of our study shows that the inhibition of PDF affects the production of Ddl enzymes. Next inhibition was of Ddl enzyme by the agrochemical which leads to the formation of the dipeptide D-alanyl-D-alanine and involves in bacterial cell wall peptidoglycan biosynthetic pathway [37]. The results of the graph reflect a little fluctuation in the production of D-Ala-D-Ala on an increasing amount of agrochemical. This graph also shows that the PDF amount remains the same in every condition. Further, the reaction was subjected to network analysis, which is one of the most important techniques for decoding the key regulatory element in reaction, essential for controlling the complex biological machinery during diseases [38]. The finding of network analysis demonstrated that Ddl and PDF enzymes are key regulatory elements, and inhibition of these enzymes through agrochemicals can be useful to protect rice crops from *Xoo*. Finally, the system biology result shows that the agrochemical affects the productivity of enzymes and will do the same effect in the bacterial cell.

The current study results suggest that using the *V*. *cinerea* plant as an agrochemical against *Xoo* might be valuable for plants and disease management and it will not affect the environment and humans. Such type of study can be used to develop new agrochemicals targeting particular enzymes to stop bacteria growth in agriculture.

## Conclusion

The overall analysis of our study indicates that methanolic extract of *V*. *cinerea* has good activity against *Xoo*. Hence, the extracts of *V*. *cinerea* can be used as pesticides against *Xoo* bacteria. Further *in silico* study showed that the compounds namely, Rutin, Methanone, [1,4-dimethyl-7-(1- methylethyl)-2-azulenyl]phenyl- have better stability with Ddl while, Phenol, 2,4-bis(1- phenylethyl)- and 1,2-Benzenedicarboxylic acid, diisooctyl ester binds favorably with PDF enzyme. This reveals the phytochemicals of *V*. *cinerea* could inhibit PDF enzymes as well as Ddl enzyme resulting ineffective growth inhibition of *Xoo*. Therefore, the present study suggests specific molecules of *V*. *cinerea* that can be utilized to develop potential natural pesticide candidates and can provide better opportunities for further innovation and development of eco-friendly antimicrobial compounds against *Xoo* infection of rice.

## Supporting information

S1 File (DOCX)
