# Supplementary material for: Antimicrobial activity of methanolic extracts of Vernonia cinerea against Xanthomonas oryzae and identification of their compounds using in silico techniques
Source: PLoS One. 2021 Jun 14;16(6):e0252759. doi: 10.1371/journal.pone.0252759 (PMC8202908; doi:10.1371/journal.pone.0252759)
Supplement: S1 File — (DOCX) [file pone.0252759.s001.docx]

**Antimicrobial activity of methanolic extracts of *Vernonia cinerea* against *Xanthomonas oryzae* and identification of their compounds using *in silico* techniques**

# Tushar Joshi^1^, Satish Chandra Pandey^1,2^, Priyanka Maiti^3^, Manish Tripathi^4^, Ashutosh Paliwal^1^, Mahesha Nand^6^, Priyanka Sharma^5^, Mukesh Samant^2^, Veena Pande^1^, Subhash Chandra^4^

1. Department of Biotechnology, Bhimtal campus, Bhimtal 263136, Kumaun University Uttarakhand, India

1. Cell in Molecular Biology laboratory, Department of Zoology, SSJ University, Almora-263601, Uttarakhand, India.
2. Centre for Environmental Assessment & Climate Change, G. B. Pant National Institute of Himalayan Environment, Kosi-Katarmal, Almora-263 643, Uttarakhand, India, priyankamaiti.06@gmail.com (P. M.)
3. Computational Biology & Biotechnology Laboratory, Department of Botany, SSJ Unversity, Almora 263601, Uttarakhand, India
4. Department of Botany, Kumaun University, D.S.B Campus, Nainital 263001, Uttarakhand, India
5. Environmental Information System on Himalayan Ecology, G.B. Pant National Institute of Himalayan Environment, Kosi‑Katarmal, Almora, Uttarakhand 263 643, India.


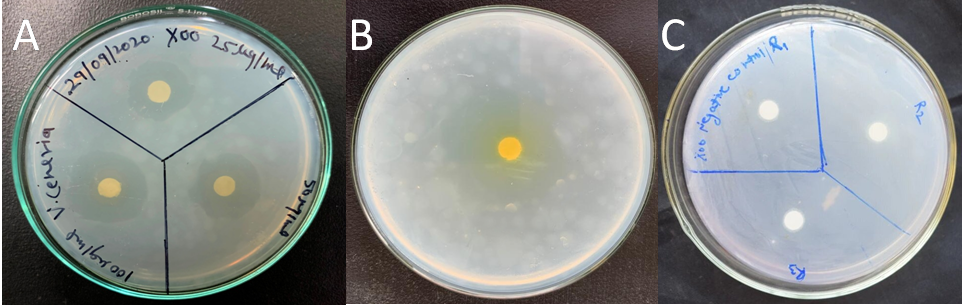


**S1 Fig -** The in vitro antibacterial activity of different concentrations of whole plant extract of V. cinerea against Xanthomonas oryzae pv oryzae (Xoo). (A) Plate (qualitative) assay using methanolic extract at the concentration of 25, 50, and 100 μg/mL (B) positive control tetracycling (1 μg/mL) (C) negative control DMSO (0.7%)


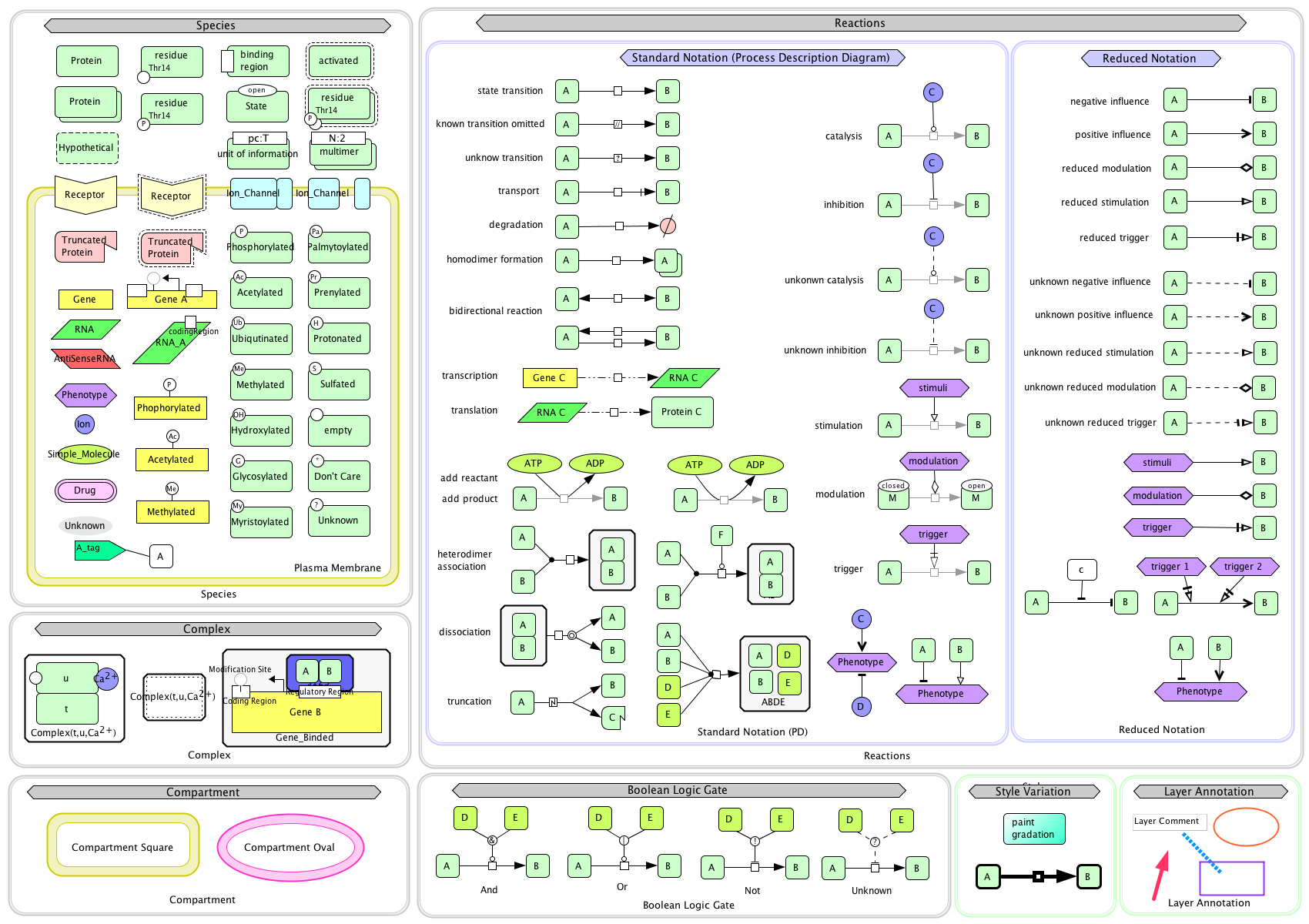


**S2 Fig** **-** System Biology Graphical Notation (SBGN) Symbols used in Cell designer 4.1 for modeling of drug inhibition activity on Ddl and PDF enzymes.

**
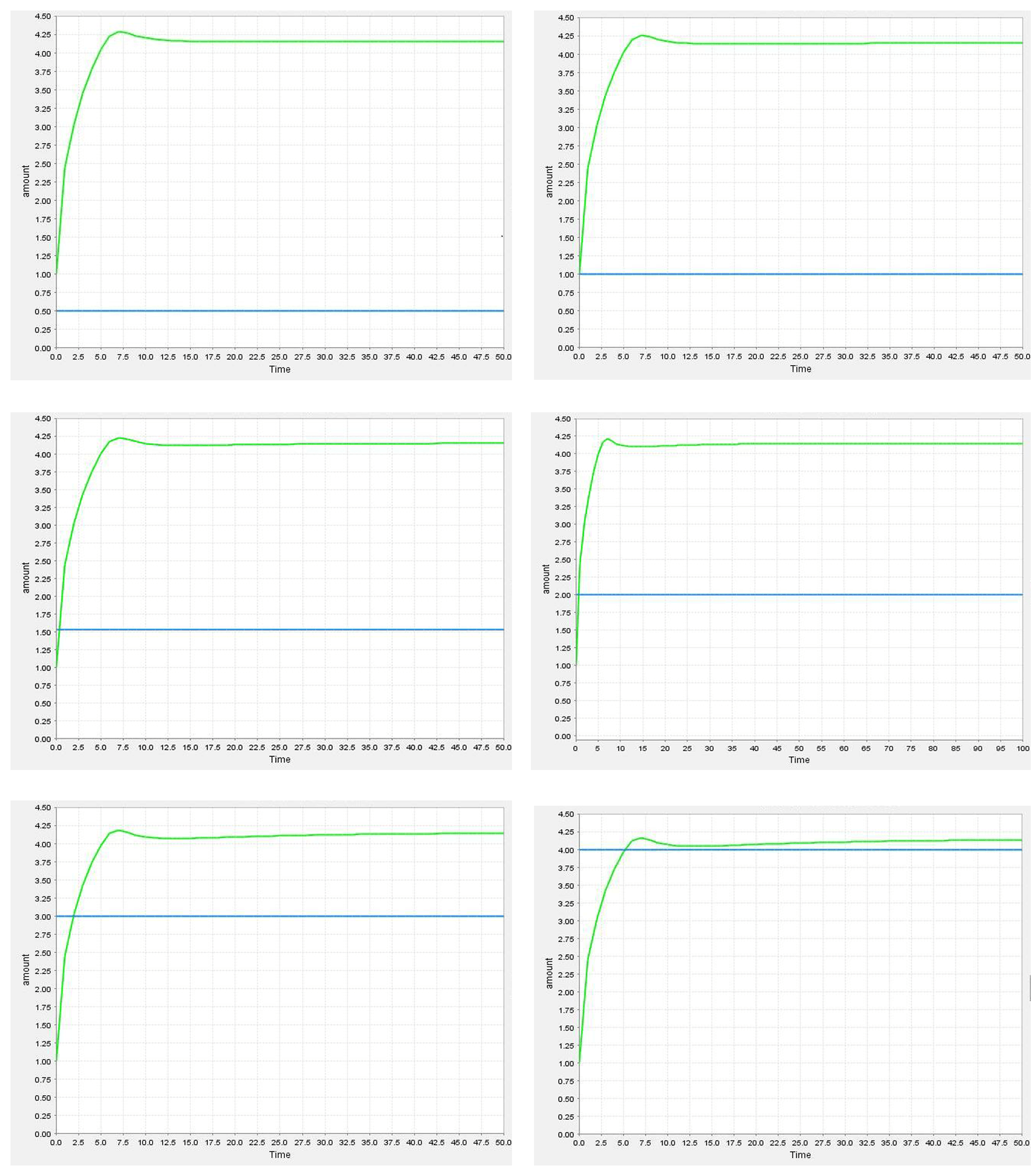
** **
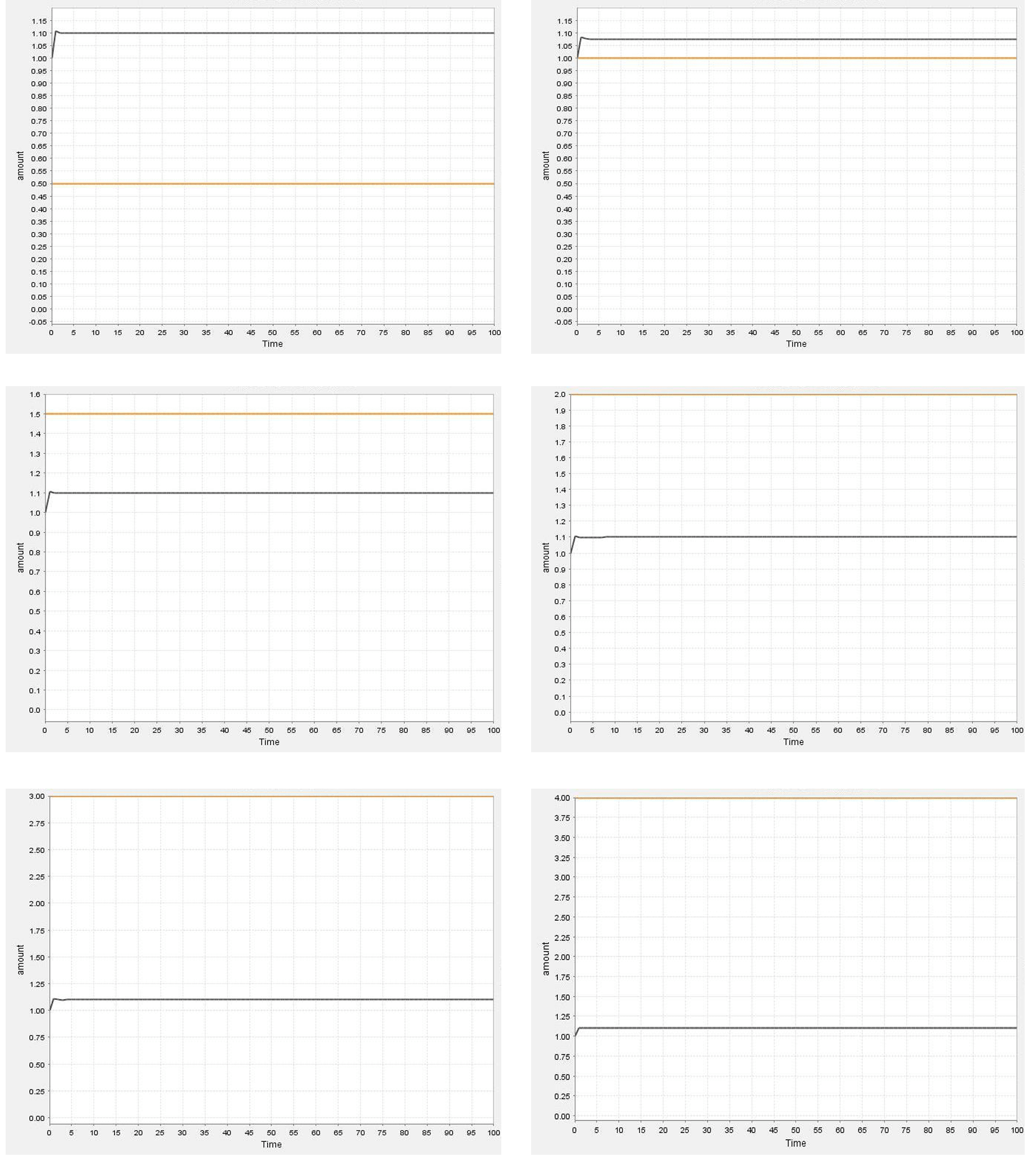
**

C

A

B

**
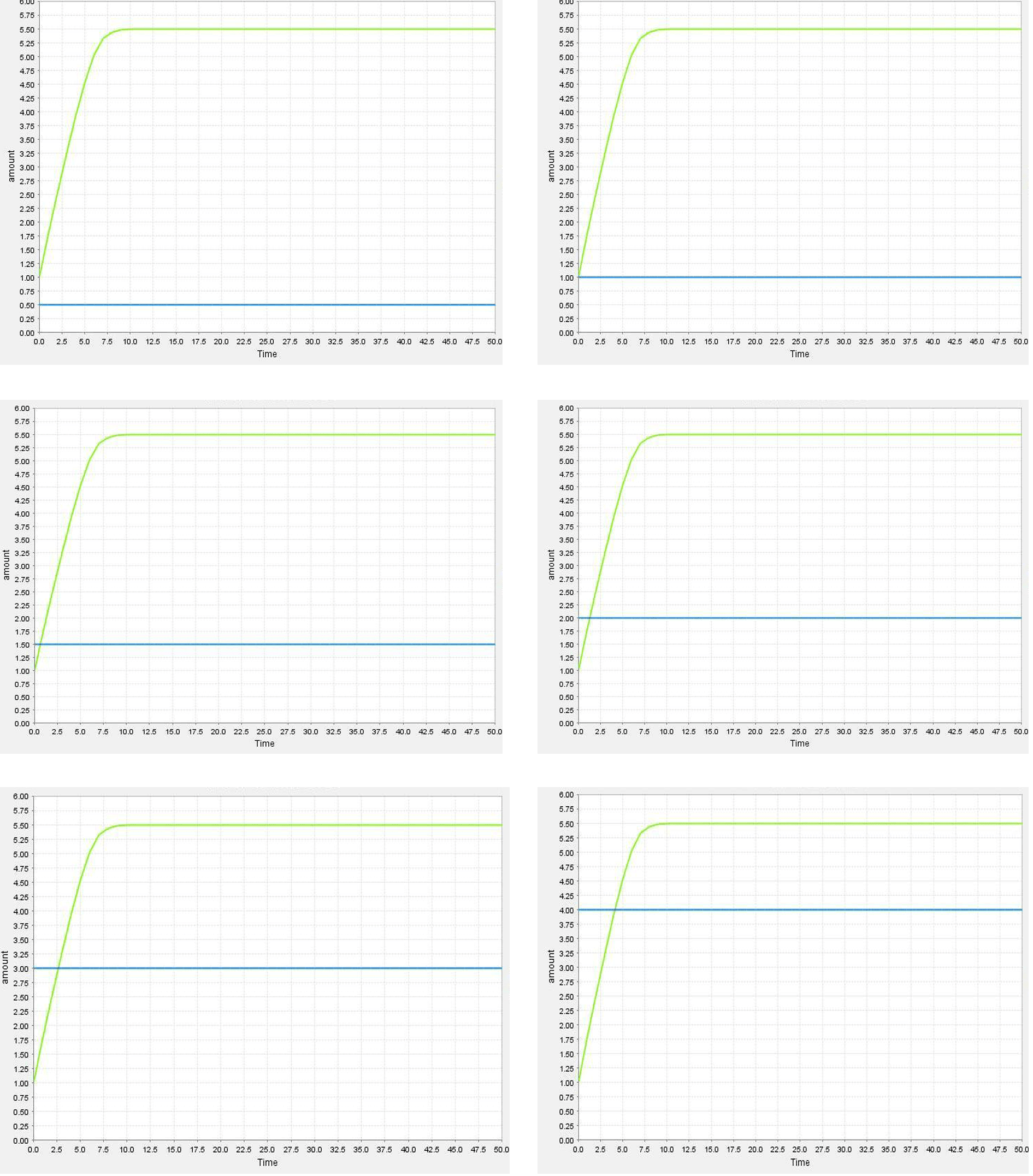
**

**S3 Fig:** Dynamic behavior analysis of drug inhibition activity on different amounts with (A) Ddl (B) D-alanyl-D-alanine and (C) PDF (amount 0.5 to 4.0).

**S1 Table:** Inhibition zones diameters (mm) and % inhibition of *V.cinerea* methanolic extracts against *Xoo*.

| **Concentration µg/ml** | **(Mean ± SD)** | |
| --- | --- | --- |
|  | **Zone of inhibition (mm)** | **% inhibition** |
| 25 | 16.0±1.0 | 31.14±0.54 |
| 50 | 18.1±1.0 | 50.50±0.89 |
| 100 | 22.6±2.08 | 74.94±0.78 |
| Tetracycline (5) | 33.1 ± 3.7 | 92.61±0.80 |

**S2 Table -** Number of class, compartment and quantity of species during simulation on cell designer

| **S.No** | **Class** | **Name** | **Compartment** | **Position to compartment** | **Included** | **Quantity type** | **Initial quantity** |
| --- | --- | --- | --- | --- | --- | --- | --- |
| **1** | Gene | ddl | c1 | inside |  | Amount | 3 |
| **2** | Rna | RNA | c1 | inside |  | Amount | 3 |
| **3** | Protein | D-ala-D-ala-ligase | c1 | inside |  | Amount | 1 |
| **4** | Simple_molecule | ATP | c1 | inside |  | Amount | 1 |
| **5** | Simple_molecule | ADP | c1 | inside |  | Amount | 1 |
| **6** | Simple_molecule | Acyl Phosphate | c1 | inside |  | Amount | 1 |
| **7** | Simple_molecule | D-Ala-D-Ala | c1 | inside |  | Amount | 1 |
| **8** | Ion | Pi | c1 | inside |  | Amount | 1 |
| **9** | Simple_molecule | D-Ala-D-Ala | c1 | transmembrane |  | Amount | 1 |
| **10** | Complex | s51 | c1 | inside | s51(s50 s49) | Amount | 6 |
| **11** | Protein | aaRS | c1 | inside |  | Amount | 3 |
| **12** | Rna | tRNAi | c1 | inside |  | Amount | 2 |
| **13** | Protein | H2N-Met | c1 | inside |  | Amount | 3 |
| **14** | Complex | s69 | c1 | inside | s69(s67 s68) | Amount | 4 |
| **15** | Protein | FMT | c1 | inside |  | Amount | 3 |
| **16** | Protein | f-met-polypeptide | c1 | inside |  | Amount | 3 |
| **17** | Protein | Met-Ppd | c1 | inside |  | Amount | 3 |
| **18** | Protein | Polypeptide | c1 | inside |  | Amount | 3 |
| **19** | Protein | MAP | c1 | inside |  | Amount | 3 |
| **20** | Protein | PDF | c1 | inside |  | Amount | 1 |
| **21** | Gene | def | c1 | inside |  | Amount | 3 |
| **22** | Drug | Drug | c1 | inside |  | Amount | 4 |
| **23** | Simple_molecule | Tetrahedral intermediate | c1 | inside |  | Amount | 1 |
| **24** | Simple_molecule | First D-Ala | c1 | inside |  | Amount | 1 |
| **25** | Simple_molecule | Second D-Ala | c1 | inside |  | Amount | 1 |

**S3 Table -** Details of reactions and kinetics rate equations used in the model

| **S.No.** | **Reaction** | **Kinetics equations** |
| --- | --- | --- |
| 1 | DNA→RNA | vmaf_re5 * (pow(s12 * c1, hic_re5_s12) / (pow(s12 * c1, hic_re5_s12) + pow(ksp_re5_s12, hic_re5_s12))) |
| 2 | RNA→D-ala-ala-ligase | vmaf_re19 * ((s13 * c1)^hic_re19_s13 / ((s13 * c1)^hic_re19_s13 + ksp_re19_s13^hic_re19_s13)) |
| 3 | D-ala-ala-ligase → inhibition  First D-ala →Acyl phosphate | kic_re23_s81 / (kic_re23_s81 + s81 * c1) * ((kcrf_re23_s23 / (kic_re23_s40_s23 * kmc_re23_s33_s23) * s23 * c1 * s40 * c1 * s33 * c1 - kcrr_re23_s23 / (kic_re23_s34_s23 * kmc_re23_s35_s23) * s23 * c1 * s35 * c1 * s34 * c1) / (1 + s40 * c1 * s33 * c1 / (kic_re23_s40_s23 * kmc_re23_s33_s23) + s40 * c1 / kic_re23_s40_s23 + s35 * c1 * s34 * c1 / (kic_re23_s34_s23 * kmc_re23_s35_s23) + s33 * c1 / kic_re23_s33_s23 + s34 * c1 / kic_re23_s34_s23 + s35 * c1 / kic_re23_s35_s23)) |
| 4 | Acyl phosphatev→ Terahedral intermediate | kass_re24 * s35 * c1 * s41 * c1 - kdiss_re24 * s39 * c1 |
| 5 | Terahedral intermediate → D-Ala-D-Ala | kass_re25 * s39 * c1 - kdiss_re25 * s42 * c1 * s43 * c1 |
| 6 | D-Ala-D-Ala → D-Ala-D-Ala | kass_re26 * s42 * c1 - kdiss_re26 * s48 * c1 |
| 7 | H_2_N-Met + tRNAi → (H_2_N-Met tRNAi) | (kcrf_re31_s55 / (kic_re31_s52_s55 * kmc_re31_s54_s55) * s55 * c1 * s52 * c1 * s54 * c1 - kcrr_re31_s55 / kmc_re31_s51_s55 * s55 * c1 * s51 * c1) / (1 + s51 * c1 / kmc_re31_s51_s55 + s52 * c1 / kic_re31_s52_s55 + s52 * c1 * s54 * c1 / (kic_re31_s52_s55 * kmc_re31_s54_s55) + s54 * c1 / kic_re31_s54_s55) |
| 8 | (H_2_N-Met tRNAi) → (OHC-NH-Met f-tRNA) | s74 * c1 * ((kcrf_re38_s74 / kmc_re38_s51_s74 * s51 * c1 - kcrr_re38_s74 / kmc_re38_s69_s74 * s69 * c1) / (1 + s51 * c1 / kmc_re38_s51_s74 + s69 * c1 / kmc_re38_s69_s74)) |
| 9 | (OHC-NH-Met f-tRNA) → f-met-polypeptide | kass_re48 * s69 * c1 - kdiss_re48 * s75 * c1 |
| 10 | f-met-polypeptide → Met-Ppd | s79 * c1 * ((kcrf_re47_s79 / kmc_re47_s75_s79 * s75 * c1 - kcrr_re47_s79 / kmc_re47_s76_s79 * s76 * c1) / (1 + s81 * c1 / kic_1_re47_s81 + (s75 * c1 / kmc_re47_s75_s79 + s76 * c1 / kmc_re47_s76_s79) * (1 + s81 * c1 / kic_2_re47_s81))) |
| 11 | Met-Ppd → Polypeptide | s78 * c1 * ((kcrf_re49_s78 / kmc_re49_s76_s78 * s76 * c1 - kcrr_re49_s78 / kmc_re49_s77_s78 * s77 * c1) / (1 + s76 * c1 / kmc_re49_s76_s78 + s77 * c1 / kmc_re49_s77_s78)) |
| 12 | Polypeptide → D-ala-ala-ligase | kass_re43 * s77 * c1 - kdiss_re43 * s23 * c1 |
| 13 | DNA→RNA | vmaf_re44 * ((s80 * c1)^hic_re44_s80 / ((s80 * c1)^hic_re44_s80 + ksp_re44_s80^hic_re44_s80)) |
| 14 | RNA → PDF | vmaf_re50 * ((s13 * c1)^hic_re50_s13 / ((s13 * c1)^hic_re50_s13 + ksp_re50_s13^hic_re50_s13)) |

**S4 Table:** Topological analysis of enzyme reaction network

| **Node** | **40** | **Edge** | **40** |
| --- | --- | --- | --- |
| **Connected component** | **1** | **Average no. of neighbors** | **1.95** |
| **Network diameter** | **19** | **Network density** | **0.050** |
| **Network radius** | **10** | **Network heterogeneity** | **0.537** |
| **Network centralization** | **0.109** | **Shortest Paths** | **1560** |
| **Characteristic Path length** | **7.721** | **Multi edge node pairs** | **1** |
